# Supplementary material for: Investigation of the Effect of C-Terminal Adjacent Phenylalanine Residues on Asparagine Deamidation by Quantum Chemical Calculations
Source: Int J Mol Sci. 2025 Jul 16;26(14):6819. doi: 10.3390/ijms26146819 (PMC12295027; doi:10.3390/ijms26146819)
Supplement: Supplementary file 1 [file ijms-26-06819-s001.zip › ijms-3709147-supplementary.pdf]

Supporting Information

Investigation of the Effect of C-terminal Adjacent Phenylalanine Residues on Asparagine Deamidation by Quantum Chemical Calculations

Koichi Kato[1,2,*], Haruka Asai,[3] Tomoki Nakayoshi[2,4], Ayato Mizuno[2], Akifumi Oda[2,5] Yoshinobu Ishikawa[1]

[1] Faculty of Pharmaceutical Sciences, Shonan University of Medical Sciences, 16-10 Kamishinano, Totsuka-ku, Yokohama, 244-0806, Japan; kato-k@kinjo-u.ac.jp (K.K.), Yoshinobu.ishikwa@sums.ac.jp (Y.I.)

[2] Faculty of Pharmacy, Meijo University, 150 Yagotoyama, Tempaku-ku, Nagoya 468-8503, Japan; 254331505@ccmailg.meijo-u.ac.jp (A.M.)

[3] Department of Pharmacology, School of Medicine, Aichi Medical University, 1-1 Yazakokarimata, Nagakute, Aichi 480-1195, Japan

[4] Institute for Advanced Research, Nagoya University, Furo-cho, Chikusa-ku, Nagoya, Aichi 464-0814, Japan

[5] Institute for Protein Research, Osaka University, 3-2 Yamadaoka, Suita 565-0871, Japan

[*] Correspondence: kato-k@kinjo-u.ac.jp (K.K.)

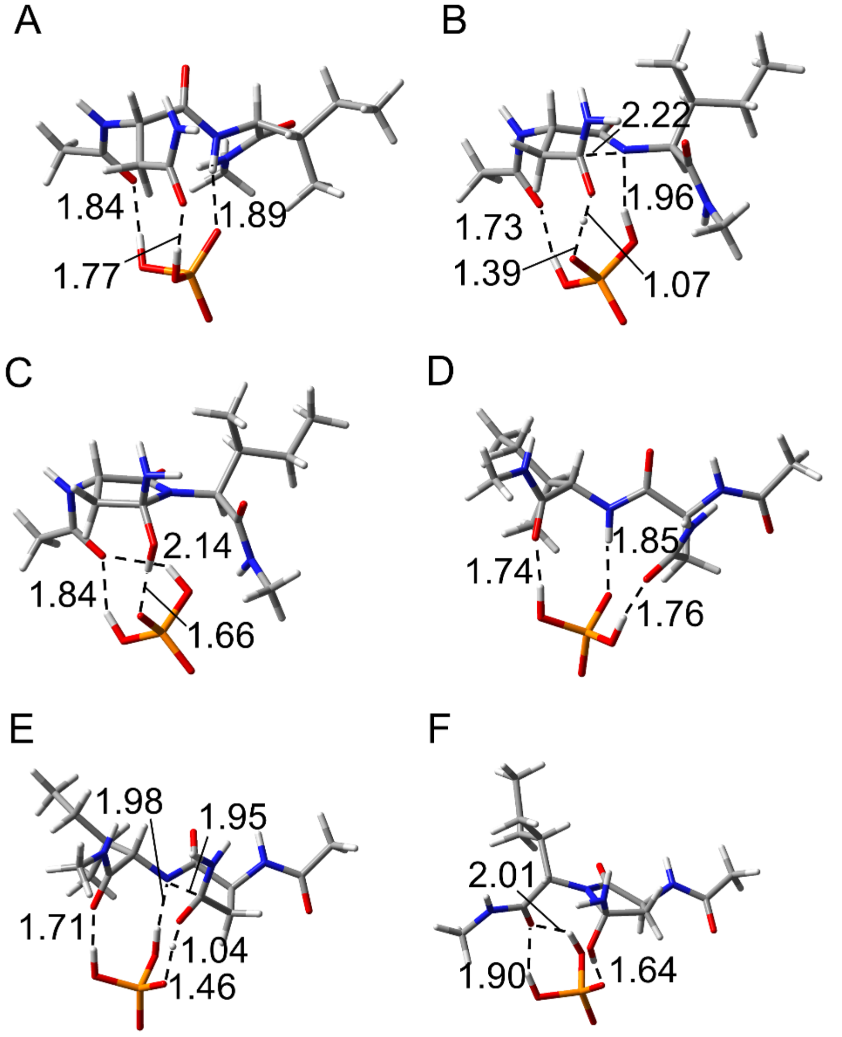

Figure S1. Optimized geometries for CH_3CO-Asn-Ile-$NHCH_3$. (A) RC, (B) TS1, and (C) INT1 for pathway 1, and (D) RC, (E) TS1, and (F) INT1 for pathway 2 were shown. Carbon, oxygen, nitrogen, phosphorus, and hydrogen atoms were illustrated in gray, red, blue, orange, and white, respectively. Selected interatomic distances are in units of Å.

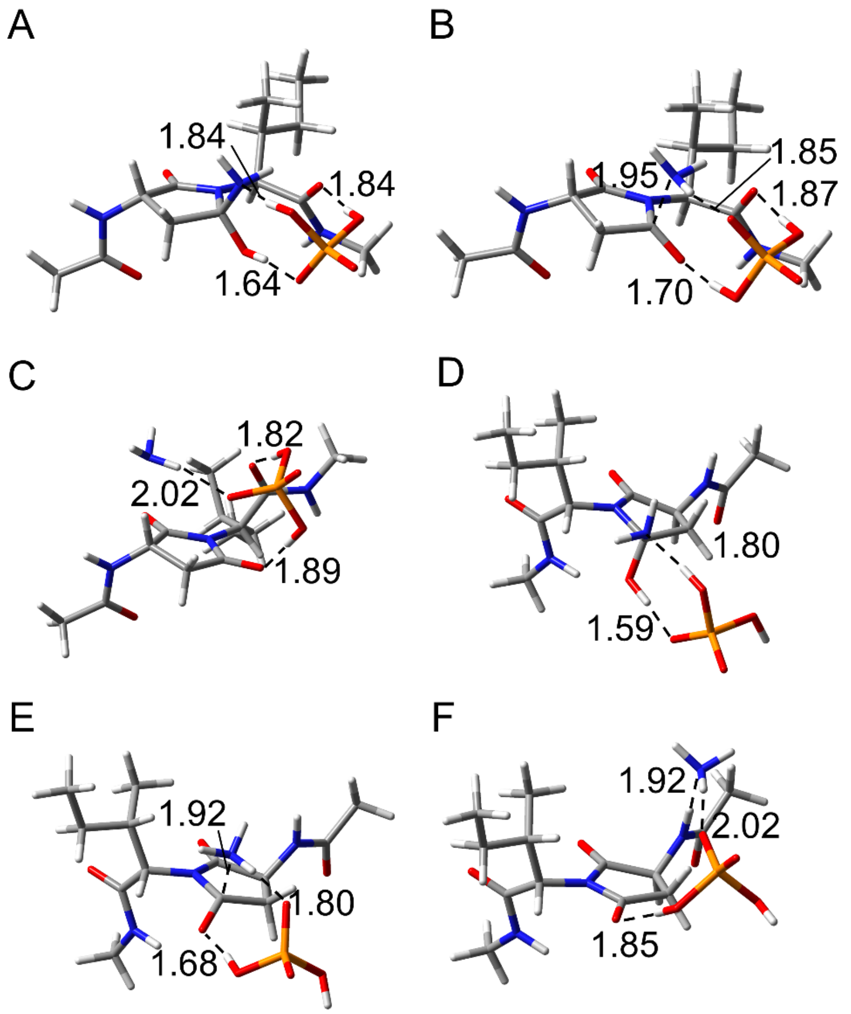

Figure S2. Optimized geometries for CH_3CO-Asn-Ile-$NHCH_3$. (A) INT2, (B) TS2, and (C) PC for pathway 1, and (D) INT2, (E) TS2, and (F) PC for pathway 2 were shown. Carbon, oxygen, nitrogen, phosphorus, and hydrogen atoms were illustrated in gray, red, blue, orange, and white, respectively. Selected interatomic distances are in units of Å.

Table S1. Dihedral angles of the optimized geometries in pathway 1 for CH_3CO-Asn-Ile-$NHCH_3$.

	**Dihedral angle**		
	φ	ψ	χ
RC	65.8	-109	-170
TS1	58.8	-147	169
INT1	52.2	-145	146
INT2	55.1	-145	143
TS2	56.5	-147	144
PC	55.7	-138	137

φ: C−N−Cα−C, ψ: N−Cα−C−N, and χ: N−Cα−Cβ−Cγ

Table S2. Dihedral angles of the optimized geometries in pathway 2 for CH_3CO-Asn-Ile-$NHCH_3$.

	**Dihedral angle**		
	φ	ψ	χ
RC	-156	-179	74.2
TS1	-168	-145	105
INT1	-165	-144	149
INT2	-113	-139	151
TS2	-117	-126	124
PC	-106	-136	134

φ: C−N−Cα−C, ψ: N−Cα−C−N, and χ: N−Cα−Cβ−Cγ

Table S3. Coordinates of all atoms in RC for CH_3CO-Asn-Phe-$NHCH_3$ in pathway 1 at B3LYP/6-31+G(d,p)

atom	coordinates		
	X	Y	Z
N	1.950857	-3.051872	-0.517506
H	1.902049	-4.06033	-0.490243
C	0.887806	-2.303065	0.154181
H	0.192384	-3.069661	0.509557
C	1.459633	-1.538249	1.367062
C	0.043212	-1.527878	-0.897964
H	2.100577	-2.242571	1.910634
H	2.099166	-0.725322	1.032466
C	0.489583	-0.962542	2.390927
O	-0.333665	-2.146645	-1.899809
C	2.933829	-2.474441	-1.243836
O	3.015364	-1.238866	-1.367728
C	3.926783	-3.400487	-1.908126
H	4.932822	-3.137105	-1.569781
H	3.743288	-4.456238	-1.699374
H	3.886541	-3.236985	-2.988988
H	0.279945	0.313499	-0.034727
P	2.999148	2.074445	0.579209
O	2.802346	1.795303	2.186616
H	2.034667	1.202984	2.377059
O	4.064826	0.898436	0.120816
O	3.71689	3.400099	0.452823
O	1.678266	1.815478	-0.12953
N	-0.523654	-1.740083	2.813743
H	-1.14763	-1.389101	3.527772
H	-0.658839	-2.687965	2.496124
O	0.669137	0.176206	2.859714
H	3.634633	0.186379	-0.39795
N	-0.343976	-0.265586	-0.603195
C	-1.187564	0.511346	-1.517232
H	-0.668734	0.610009	-2.479741
C	-2.571529	-0.137965	-1.848365
C	-1.399008	1.90887	-0.914228
H	-2.362551	-1.059999	-2.392432
O	-1.59255	2.08433	0.293535
N	-1.435713	2.915942	-1.814271
H	-1.196509	2.72377	-2.776499
C	-1.703564	4.295931	-1.429619
H	-2.643592	4.362786	-0.875333
H	-0.90107	4.694287	-0.800113
H	-1.779423	4.899117	-2.334669
H	-3.061278	0.546826	-2.549123
C	-3.507424	-0.430132	-0.694293
C	-4.48441	0.49898	-0.302191
C	-3.454137	-1.660182	-0.019409
C	-5.369737	0.217541	0.742234
H	-4.555874	1.450935	-0.822052
C	-4.335642	-1.946794	1.02748
H	-2.72524	-2.405337	-0.325622
C	-5.29601	-1.00689	1.414554
H	-6.118951	0.951229	1.026575
H	-4.277046	-2.906276	1.534032
H	-5.98498	-1.229384	2.224357

Table S4. Coordinates of all atoms in TS1 for CH_3CO-Asn-Phe-$NHCH_3$ in pathway 1 at B3LYP/6-31+G(d,p)

atom	coordinates		
	X	Y	Z
N	1.395956	-3.246473	0.036492
H	1.129513	-4.218572	0.098578
C	0.388587	-2.244489	0.363239
H	-0.529279	-2.815177	0.541155
C	0.742206	-1.442653	1.627825
C	0.034587	-1.290259	-0.80416
H	0.619237	-2.062368	2.522929
H	1.783664	-1.12584	1.573839
C	-0.109881	-0.199519	1.795273
O	-0.031367	-1.730082	-1.975837
C	2.63558	-2.9555	-0.410695
O	3.011922	-1.777951	-0.551757
C	3.542788	-4.120887	-0.73303
H	4.453898	-4.034835	-0.13418
H	3.081066	-5.092209	-0.544652
H	3.830632	-4.059842	-1.786619
H	1.483281	0.883226	-0.597434
P	3.417638	1.598362	0.222908
O	2.866522	1.145167	1.589467
H	1.484441	0.989345	1.888248
O	4.473549	0.47601	-0.308411
O	4.089445	2.940322	0.111152
O	2.17824	1.535023	-0.846058
N	-1.402517	-0.309848	2.199033
H	-1.91957	0.554343	2.038937
H	-1.901288	-1.121409	1.854649
O	0.464041	0.92029	2.144633
H	4.057028	-0.414471	-0.374754
N	-0.275142	-0.079031	-0.342698
C	-1.015594	0.840516	-1.205613
H	-0.39895	1.093317	-2.081229
C	-2.349793	0.27964	-1.82114
C	-1.318272	2.144628	-0.450574
H	-2.050203	-0.476636	-2.549331
O	-1.902183	2.172444	0.64379
N	-0.971802	3.279666	-1.098662
H	-0.448299	3.208547	-1.958957
C	-1.245938	4.606516	-0.562415
H	-2.316533	4.734711	-0.378325
H	-0.71182	4.770415	0.379143
H	-0.918411	5.348718	-1.291018
H	-2.8073	1.105919	-2.377996
C	-3.368397	-0.320158	-0.876266
C	-4.394428	0.456818	-0.314315
C	-3.337658	-1.691518	-0.570653
C	-5.341556	-0.108654	0.544109
H	-4.450564	1.515654	-0.549309
C	-4.279232	-2.262723	0.292532
H	-2.578008	-2.319559	-1.027836
C	-5.283743	-1.470946	0.858011
H	-6.126228	0.513915	0.965332
H	-4.233921	-3.325885	0.51257
H	-6.019296	-1.911592	1.524894

Table S5. Coordinates of all atoms in INT1 in for CH_3CO-Asn-Phe-$NHCH_3$ pathway 1 at B3LYP/6-31+G(d,p)

atom	coordinates		
	X	Y	Z
N	-1.099067	3.365854	0.220259
H	-0.893779	4.34817	0.334115
C	-0.023166	2.421829	0.480284
H	0.886536	3.024349	0.563384
C	-0.192107	1.525931	1.716346
C	0.221522	1.433238	-0.667453
H	0.244679	1.956425	2.618625
H	-1.253278	1.342983	1.892251
C	0.483672	0.170461	1.380001
O	0.184423	1.712651	-1.869418
C	-2.344528	3.017744	-0.161284
O	-2.659931	1.82414	-0.342221
C	-3.343109	4.133615	-0.35433
H	-4.18851	3.969111	0.320108
H	-2.923357	5.123431	-0.165509
H	-3.722098	4.092825	-1.379388
H	-2.238558	-0.083383	-1.128061
P	-3.641726	-1.235468	0.093723
O	-2.857619	-1.052872	1.389917
H	-1.214824	-0.884515	1.55108
O	-4.699242	0.005126	-0.081725
O	-4.374912	-2.523593	-0.171048
O	-2.594153	-0.990857	-1.175134
N	1.780383	0.032194	2.00573
H	2.186947	-0.858601	1.7205
H	2.41043	0.764503	1.687745
O	-0.247105	-0.936341	1.795205
H	-4.204956	0.854974	-0.094006
N	0.554061	0.230528	-0.138314
C	0.981533	-0.853365	-1.039699
H	0.252964	-0.852809	-1.856347
C	2.360984	-0.610721	-1.737711
C	0.910503	-2.243158	-0.373613
H	2.168088	0.110201	-2.536169
O	1.851368	-2.708965	0.28411
N	-0.209846	-2.940162	-0.654626
H	-1.00006	-2.428656	-1.038732
C	-0.467062	-4.243964	-0.058058
H	0.370028	-4.918166	-0.253591
H	-0.605201	-4.168364	1.026848
H	-1.370797	-4.658921	-0.505829
H	2.633555	-1.554981	-2.220539
C	3.523765	-0.100467	-0.91104
C	4.43887	-0.971931	-0.298874
C	3.739103	1.282037	-0.783306
C	5.519931	-0.478051	0.4369
H	4.292178	-2.042829	-0.390702
C	4.815336	1.781746	-0.042095
H	3.063941	1.975032	-1.278642
C	5.709295	0.901549	0.574798
H	6.215848	-1.171236	0.901508
H	4.958699	2.855275	0.04393
H	6.548809	1.285025	1.14754

Table S6. Coordinates of all atoms in INT2 for CH_3CO-Asn-Phe-$NHCH_3$ in pathway 1 at B3LYP/6-31+G(d,p)

atom	coordinates		
	X	Y	Z
N	4.210476	-0.771027	-1.151994
H	4.865498	-0.450548	-1.850023
C	2.892671	-0.162779	-1.134962
H	2.929407	0.653817	-1.862438
C	1.710571	-1.099782	-1.43553
C	2.528302	0.485148	0.20864
H	1.406054	-1.066383	-2.483741
H	1.973886	-2.125367	-1.180028
C	0.547955	-0.668274	-0.501116
O	3.293362	1.140462	0.919155
C	4.616355	-1.717784	-0.265326
O	3.870837	-2.134249	0.631897
C	6.028698	-2.237595	-0.437347
H	6.5884	-2.045362	0.482621
H	5.989547	-3.320995	-0.582366
H	6.559215	-1.784829	-1.27782
H	-3.145576	-0.670651	0.640739
P	-3.371653	-2.229746	-0.930982
O	-2.152585	-2.954193	-0.351562
H	-0.775848	-2.128741	-0.063761
O	-4.585349	-3.012388	-1.365225
O	-2.858379	-1.334348	-2.203063
O	-3.882608	-1.118916	0.170621
N	-0.541614	-0.055346	-1.262426
H	-1.128915	0.472494	-0.612763
H	-0.164632	0.607934	-1.936095
O	0.074194	-1.721441	0.282443
H	-1.963336	-0.944564	-2.002042
N	1.204004	0.289756	0.438301
C	0.590788	0.86518	1.648486
H	1.286993	0.647861	2.464739
C	0.486177	2.428649	1.607158
C	-0.769184	0.245584	2.012453
H	1.511462	2.796135	1.693918
O	-1.781145	0.392236	1.298904
N	-0.823868	-0.349988	3.213419
H	0.031532	-0.479567	3.734754
C	-2.053353	-0.919464	3.754665
H	-2.349458	-1.816219	3.201286
H	-1.881031	-1.182943	4.79818
H	-2.86297	-0.188276	3.69616
H	-0.038576	2.73019	2.52039
C	-0.164996	3.068088	0.39888
C	-1.53011	3.396006	0.386763
C	0.604598	3.390803	-0.731207
C	-2.113462	4.006256	-0.727248
H	-2.141138	3.168024	1.254721
C	0.025135	3.996391	-1.850633
H	1.670621	3.179947	-0.73034
C	-1.339018	4.30315	-1.853841
H	-3.171677	4.251945	-0.713861
H	0.6406	4.236025	-2.713244
H	-1.791622	4.777733	-2.719726

Table S7. Coordinates of all atoms in TS2 for CH_3CO-Asn-Phe-$NHCH_3$ in pathway 1 at B3LYP/6-31+G(d,p)

atom	coordinates		
	X	Y	Z
N	4.088289	-0.90013	-1.048757
H	4.744075	-0.678471	-1.7835
C	2.7772	-0.282853	-1.104621
H	2.815309	0.434141	-1.930111
C	1.586077	-1.243675	-1.275415
C	2.429665	0.529033	0.153154
H	1.264408	-1.33115	-2.315481
H	1.847763	-2.234514	-0.906819
C	0.454942	-0.705769	-0.35775
O	3.205592	1.247348	0.780229
C	4.485595	-1.724353	-0.042871
O	3.733487	-2.008132	0.89914
C	5.893123	-2.273868	-0.140609
H	6.462898	-1.937264	0.730543
H	5.847737	-3.365966	-0.10661
H	6.416884	-1.966462	-1.048299
H	-3.140361	-0.908956	0.555541
P	-3.007264	-2.568064	-0.94196
O	-1.7787	-3.196944	-0.185749
H	-0.857235	-2.35197	0.104136
O	-4.069523	-3.570871	-1.350097
O	-2.477213	-1.637695	-2.076028
O	-3.749339	-1.570828	0.175955
N	-0.645755	-0.05906	-1.206308
H	-1.224135	0.501285	-0.557558
H	-0.261259	0.560734	-1.920381
O	-0.082664	-1.610711	0.490795
H	-1.358859	-0.793416	-1.658952
N	1.099441	0.379954	0.413671
C	0.501391	1.051806	1.584274
H	1.169216	0.849231	2.427527
C	0.470822	2.612616	1.440855
C	-0.900084	0.53182	1.950102
H	1.511982	2.938447	1.491704
O	-1.872518	0.608982	1.170097
N	-1.039633	0.120304	3.217956
H	-0.216758	0.031833	3.797084
C	-2.320667	-0.310236	3.768856
H	-2.644483	-1.253921	3.319062
H	-2.204152	-0.446912	4.843778
H	-3.084709	0.447856	3.581747
H	-0.029083	2.996478	2.336715
C	-0.172723	3.191801	0.198505
C	-1.518382	3.592303	0.189503
C	0.583633	3.379759	-0.97072
C	-2.097468	4.142745	-0.957331
H	-2.117745	3.470667	1.086791
C	0.00756	3.924899	-2.122722
H	1.637228	3.114518	-0.97502
C	-1.337992	4.305134	-2.120743
H	-3.140253	4.446856	-0.940774
H	0.612138	4.061242	-3.014925
H	-1.787173	4.733051	-3.012261

Table S8. Coordinates of all atoms in INT3 for CH_3CO-Asn-Phe-$NHCH_3$ in pathway 1 at B3LYP/6-31+G(d,p)

atom	coordinates		
	X	Y	Z
N	4.105509	-1.180821	-1.003766
H	4.777842	-0.97137	-1.727026
C	2.811642	-0.526037	-1.075899
H	2.879206	0.182111	-1.907586
C	1.597448	-1.454663	-1.247761
C	2.477633	0.306757	0.173438
H	1.304467	-1.57775	-2.293209
H	1.81628	-2.43657	-0.829869
C	0.450436	-0.859393	-0.383128
O	3.288278	0.970353	0.823022
C	4.472397	-2.01811	0.002044
O	3.706846	-2.29283	0.936424
C	5.86911	-2.5985	-0.084242
H	6.438786	-2.274556	0.791839
H	5.799823	-3.689369	-0.050862
H	6.407807	-2.302678	-0.987095
H	-3.183297	-0.467755	0.416525
P	-3.407544	-2.235049	-0.938728
O	-2.336712	-3.098251	-0.07367
H	-1.474835	-2.574798	0.053426
O	-4.619284	-3.088314	-1.229677
O	-2.663323	-1.572549	-2.107448
O	-3.905067	-1.054191	0.103517
N	-0.539043	-0.073694	-1.346576
H	-1.078883	0.561845	-0.738413
H	-0.066701	0.469811	-2.069577
O	-0.225596	-1.683197	0.36915
H	-1.253612	-0.732157	-1.768272
N	1.138833	0.236628	0.395431
C	0.549058	0.899272	1.56929
H	1.131891	0.583722	2.441636
C	0.677235	2.461879	1.524217
C	-0.919472	0.517284	1.813939
H	1.741006	2.678015	1.640653
O	-1.817946	0.732142	0.970751
N	-1.198344	0.096123	3.055485
H	-0.433357	-0.104025	3.683892
C	-2.555319	-0.187864	3.510911
H	-2.954704	-1.085113	3.028517
H	-2.530371	-0.341713	4.58966
H	-3.211964	0.654779	3.282093
H	0.171841	2.839221	2.419883
C	0.153574	3.176402	0.295876
C	-1.135969	3.731144	0.267309
C	0.971403	3.341136	-0.83466
C	-1.602195	4.412481	-0.860431
H	-1.78099	3.627383	1.134927
C	0.507904	4.016932	-1.967961
H	1.985996	2.953172	-0.821474
C	-0.783376	4.552936	-1.985906
H	-2.603008	4.835194	-0.858655
H	1.158789	4.13256	-2.830123
H	-1.144153	5.082874	-2.86275

Table S9. Coordinates of all atoms in TS3 for CH_3CO-Asn-Phe-$NHCH_3$ in pathway 1 at B3LYP/6-31+G(d,p)

atom	coordinates		
	X	Y	Z
N	4.071127	-1.353724	-1.033233
H	4.686963	-1.15659	-1.808367
C	2.751637	-0.752371	-1.048466
H	2.719485	-0.105204	-1.93043
C	1.563537	-1.726018	-1.050095
C	2.481823	0.157323	0.164422
H	1.197746	-1.971249	-2.047151
H	1.8367	-2.649918	-0.536165
C	0.492149	-1.050044	-0.190333
O	3.312803	0.874031	0.715236
C	4.539315	-2.107471	-0.002947
O	3.84578	-2.349652	0.994364
C	5.950188	-2.638589	-0.145863
H	5.924028	-3.729882	-0.077724
H	6.429711	-2.351523	-1.084199
H	6.551038	-2.267687	0.689565
H	-3.184815	-0.241806	0.341268
P	-3.663875	-2.008779	-0.953914
O	-2.671923	-2.989766	-0.092481
H	-1.779123	-2.576834	0.034109
O	-4.972684	-2.738516	-1.153376
O	-2.898658	-1.476448	-2.163087
O	-3.97591	-0.764682	0.081733
N	-0.490634	-0.031264	-1.560524
H	-0.9149	0.722785	-1.01921
H	0.051252	0.363812	-2.326336
O	-0.378921	-1.695826	0.437265
H	-1.261012	-0.61971	-1.920564
N	1.154575	0.062836	0.485822
C	0.570681	0.805038	1.615897
H	1.087241	0.467443	2.521386
C	0.809587	2.353281	1.553802
C	-0.935119	0.537521	1.792287
H	1.88032	2.504228	1.697328
O	-1.768991	0.815829	0.910269
N	-1.293779	0.138806	3.024461
H	-0.572183	-0.128474	3.678807
C	-2.684907	-0.041016	3.425427
H	-3.129971	-0.913273	2.937257
H	-2.716284	-0.179295	4.506231
H	-3.268498	0.843361	3.158954
H	0.306233	2.763854	2.435898
C	0.360856	3.104958	0.31623
C	-0.889158	3.742923	0.267492
C	1.213725	3.233477	-0.792627
C	-1.285385	4.467698	-0.859654
H	-1.558795	3.669911	1.119301
C	0.819875	3.953076	-1.925492
H	2.200939	2.782122	-0.762831
C	-0.433663	4.57065	-1.964706
H	-2.256863	4.953926	-0.87322
H	1.496769	4.038556	-2.771049
H	-0.739701	5.134183	-2.841445

Table S10. Coordinates of all atoms in PC for CH_3CO-Asn-Phe-$NHCH_3$ in pathway 1 at B3LYP/6-31+G(d,p)

atom	coordinates		
	X	Y	Z
N	3.977378	-0.705632	0.689254
H	4.412737	-0.73265	1.599998
C	2.608006	-0.245874	0.602691
H	2.243883	-0.083453	1.62813
C	2.360404	1.026549	-0.221993
C	1.665996	-1.2911	-0.010829
H	2.138837	1.903479	0.393211
H	3.189034	1.254905	-0.896793
C	1.140167	0.705497	-1.040373
O	1.695811	-2.487351	0.205116
C	4.672762	-1.139722	-0.394644
O	4.158122	-1.153469	-1.522092
C	6.093645	-1.598992	-0.152089
H	6.765501	-1.017027	-0.789169
H	6.409657	-1.496528	0.888201
H	6.179323	-2.648301	-0.449269
H	-2.001036	2.084299	0.325651
P	-0.754655	3.906228	0.630016
O	-0.302835	4.023187	-0.954316
H	0.092643	3.187787	-1.278864
O	-1.147811	5.297923	1.070581
O	0.310547	3.121267	1.381238
O	-2.144021	3.026744	0.568203
N	1.047363	0.900838	3.340071
H	0.236005	0.37023	3.650106
H	1.445282	1.33106	4.172261
O	0.55089	1.454399	-1.814073
H	0.707362	1.657512	2.737863
N	0.740796	-0.609712	-0.8147
C	-0.441682	-1.113616	-1.542957
H	-0.186625	-1.037824	-2.604809
C	-0.856315	-2.598648	-1.29008
C	-1.626127	-0.165176	-1.253688
H	0.049791	-3.202101	-1.244435
O	-1.777618	0.355638	-0.137365
N	-2.501836	-0.020921	-2.260024
H	-2.275822	-0.409673	-3.165259
C	-3.724838	0.767151	-2.138755
H	-3.50588	1.839389	-2.126336
H	-4.36588	0.542619	-2.991344
H	-4.247015	0.504727	-1.216179
H	-1.388584	-2.893027	-2.199381
C	-1.760909	-2.896778	-0.103902
C	-3.150894	-2.965379	-0.29645
C	-1.256877	-3.15133	1.181703
C	-4.015025	-3.258391	0.762737
H	-3.562522	-2.792846	-1.287573
C	-2.117977	-3.44633	2.24335
H	-0.186133	-3.125885	1.349223
C	-3.500637	-3.496793	2.040544
H	-5.086056	-3.306001	0.587013
H	-1.705527	-3.641596	3.22943
H	-4.167944	-3.727636	2.865934

Table S11. Coordinates of all atoms in RC for CH_3CO-Asn-Phe-$NHCH_3$ in pathway 2 at B3LYP/6-31+G(d,p)

atom	coordinates		
	X	Y	Z
N	-3.609056	-1.909801	-0.347017
H	-3.199141	-2.713335	0.121849
C	-2.649446	-0.867234	-0.689775
H	-2.619467	-0.766814	-1.782032
C	-3.039187	0.531713	-0.148749
C	-1.258841	-1.363336	-0.230633
H	-2.419755	1.282779	-0.641676
H	-4.078317	0.713067	-0.443581
C	-2.915704	0.753617	1.350708
O	-1.138269	-2.493143	0.271663
O	-2.341635	1.760118	1.810146
N	-3.473749	-0.163924	2.161632
H	-3.422172	-0.042377	3.1641
H	-3.952963	-0.971611	1.787801
N	-0.226354	-0.525071	-0.438941
H	-0.367015	0.434241	-0.807829
C	1.142935	-0.920786	-0.112151
H	1.161413	-2.01221	-0.082937
C	2.117899	-0.423337	-1.204427
C	1.523954	-0.374583	1.275953
H	1.729525	-0.786828	-2.161911
O	1.600254	0.848106	1.503823
C	-4.793589	-2.050606	-1.005949
O	-5.199342	-1.204111	-1.813503
C	-5.59914	-3.289185	-0.673576
H	-5.127286	-3.920851	0.082024
H	-6.587	-2.983438	-0.317536
H	-5.741804	-3.871358	-1.588831
N	1.763688	-1.288024	2.228171
H	1.659165	-2.266177	1.997914
C	2.129712	-0.929238	3.594855
H	3.03644	-0.31857	3.600653
H	1.325809	-0.365219	4.076874
H	2.310794	-1.846681	4.154356
P	-0.050674	3.416108	-0.732884
O	-1.179615	3.789733	0.395361
O	0.160614	4.651819	-1.575979
O	-0.44041	2.095609	-1.400878
O	1.322454	3.183151	0.131202
H	-1.566187	3.002337	0.854657
H	1.367369	2.296014	0.564659
H	2.090578	0.669083	-1.235776
C	3.539633	-0.909042	-1.00478
C	4.527646	-0.060191	-0.483803
C	3.896435	-2.226785	-1.335504
C	5.837084	-0.514735	-0.29384
H	4.271435	0.964685	-0.22906
C	5.20275	-2.685442	-1.146487
H	3.147734	-2.8966	-1.752178
C	6.178449	-1.829722	-0.6229
H	6.588405	0.159456	0.107636
H	5.459738	-3.706871	-1.412593
H	7.195174	-2.183516	-0.478563

Table S12. Coordinates of all atoms in TS1 for CH_3CO-Asn-Phe-$NHCH_3$ in pathway 2 at B3LYP/6-31+G(d,p)

atom	coordinates		
	X	Y	Z
N	-3.549612	-1.970917	-0.338047
H	-3.046067	-2.847663	-0.412066
C	-2.761779	-0.767274	-0.585465
H	-3.054937	-0.344853	-1.554627
C	-2.877544	0.316858	0.501221
C	-1.280861	-1.184462	-0.653455
H	-2.863424	1.306434	0.042543
H	-3.806929	0.223356	1.067524
C	-1.690731	0.23095	1.456141
O	-0.939495	-2.24257	-1.211544
O	-1.181039	1.339917	1.967326
N	-1.736096	-0.783754	2.384819
H	-0.947811	-0.838701	3.018083
H	-2.084965	-1.6825	2.07875
N	-0.505715	-0.302393	0.009597
H	-0.589006	1.274279	-1.183662
C	0.89885	-0.700842	0.201585
H	0.928682	-1.754479	0.508785
C	1.722733	-0.568534	-1.112089
C	1.555716	0.152161	1.299906
H	1.157822	-1.080301	-1.894485
O	1.828596	1.356615	1.144824
C	-4.904576	-1.988157	-0.458953
O	-5.558882	-0.952143	-0.641562
C	-5.570682	-3.342953	-0.33393
H	-4.865051	-4.155348	-0.14712
H	-6.295824	-3.305739	0.483976
H	-6.120836	-3.551335	-1.256281
N	1.861355	-0.490348	2.440362
H	1.695483	-1.485454	2.495989
C	2.538586	0.164255	3.555717
H	3.531934	0.513053	3.257788
H	1.957569	1.0222	3.903266
H	2.63855	-0.555457	4.367959
P	-0.361296	3.444052	-0.879315
O	-1.167945	3.316836	0.424881
O	-0.607509	4.628413	-1.773596
O	-0.616176	2.077647	-1.754036
O	1.228916	3.390101	-0.521949
H	-1.174829	2.136307	1.298726
H	1.450729	2.626071	0.069898
H	1.786182	0.489548	-1.378229
C	3.112176	-1.165088	-1.011265
C	4.243606	-0.350316	-0.851834
C	3.296488	-2.556852	-1.07415
C	5.523413	-0.907482	-0.756225
H	4.121701	0.728533	-0.806863
C	4.572565	-3.118039	-0.977088
H	2.434007	-3.20493	-1.209816
C	5.69212	-2.293883	-0.816359
H	6.386245	-0.25803	-0.637466
H	4.693386	-4.196305	-1.032827
H	6.685112	-2.728167	-0.744511

Table S13. Coordinates of all atoms in INT1 for CH_3CO-Asn-Phe-$NHCH_3$ in pathway 2 at B3LYP/6-31+G(d,p)

atom	coordinates		
	X	Y	Z
N	-3.860906	-1.69745	-0.435009
H	-3.478748	-2.633411	-0.479482
C	-2.926908	-0.581177	-0.517018
H	-3.147094	-0.01043	-1.426601
C	-2.877554	0.346536	0.706261
C	-1.504023	-1.132135	-0.635463
H	-2.909363	1.390067	0.388071
H	-3.700398	0.169764	1.399104
C	-1.522473	0.100243	1.412772
O	-1.186453	-2.034406	-1.416632
O	-0.928889	1.234783	1.923529
N	-1.668101	-0.805973	2.530299
H	-0.786789	-0.915767	3.024327
H	-1.998941	-1.722658	2.242829
N	-0.693578	-0.498802	0.256395
H	-0.418291	2.051182	-2.04377
C	0.725158	-0.916243	0.343247
H	0.742665	-1.991309	0.561297
C	1.469227	-0.68194	-1.001069
C	1.507302	-0.178693	1.453066
H	0.838959	-1.065894	-1.803159
O	1.94653	0.973032	1.309407
C	-5.19763	-1.527804	-0.608989
O	-5.700207	-0.402971	-0.742569
C	-6.040311	-2.786539	-0.613729
H	-5.455675	-3.699467	-0.480953
H	-6.780466	-2.718258	0.188678
H	-6.582488	-2.84363	-1.562004
N	1.759595	-0.895176	2.563383
H	1.492062	-1.869554	2.586965
C	2.562156	-0.372479	3.665743
H	3.588392	-0.173729	3.342015
H	2.128138	0.557253	4.041169
H	2.572958	-1.113134	4.465072
P	0.233429	3.70732	-0.747575
O	-0.845411	3.223545	0.220558
O	0.363066	5.168523	-1.090092
O	0.007611	2.909588	-2.183123
O	1.692328	3.187268	-0.23607
H	-0.87224	1.957696	1.230872
H	1.674384	2.340719	0.274693
H	1.589927	0.393595	-1.145904
C	2.819742	-1.370503	-1.050895
C	4.013404	-0.644198	-0.9248
C	2.900982	-2.761245	-1.236095
C	5.254816	-1.28702	-0.980287
H	3.969956	0.432084	-0.784258
C	4.138585	-3.407876	-1.288831
H	1.98818	-3.340922	-1.350248
C	5.321623	-2.671673	-1.159868
H	6.167208	-0.705095	-0.884274
H	4.179326	-4.483436	-1.436464
H	6.284658	-3.172082	-1.204153

Table S14. Coordinates of all atoms in INT2 for CH_3CO-Asn-Phe-$NHCH_3$ in pathway 2 at B3LYP/6-31+G(d,p)

atom	coordinates		
	X	Y	Z
N	-4.110816	-1.11358	0.371826
H	-3.843745	-1.128116	1.346646
C	-3.098554	-1.373044	-0.649168
H	-3.452503	-2.196381	-1.275892
C	-2.685923	-0.165119	-1.50074
C	-1.786935	-1.798376	0.017405
H	-2.45444	-0.499657	-2.515052
H	-3.458152	0.603374	-1.557892
C	-1.376347	0.384637	-0.884449
O	-1.618542	-2.858409	0.621871
O	-0.538006	0.823945	-1.891995
N	-1.576267	1.470975	0.095631
H	-2.053259	1.120152	0.924002
H	-2.20496	2.156199	-0.32412
N	-0.849482	-0.821879	-0.16448
H	2.505094	2.389165	0.531664
C	0.548542	-1.095482	0.207246
H	0.475222	-2.008352	0.807119
C	1.447705	-1.40268	-1.014485
C	1.146706	-0.010909	1.127853
H	0.832113	-1.966603	-1.722743
O	2.104201	0.691382	0.765475
C	-5.435313	-1.066884	0.063818
O	-5.83444	-1.16541	-1.10411
C	-6.388593	-0.875668	1.224793
H	-5.886074	-0.810546	2.19234
H	-6.964003	0.039865	1.060464
H	-7.092612	-1.712486	1.242878
N	0.602081	0.083564	2.352082
H	-0.160347	-0.533625	2.595652
C	1.047714	1.059787	3.342213
H	2.115936	0.939531	3.54305
H	0.868055	2.079208	2.989246
H	0.489816	0.893152	4.263521
P	1.230874	3.901909	-0.396351
O	0.873686	3.018636	-1.59584
O	1.418286	5.386636	-0.593459
O	2.604999	3.340918	0.302302
O	0.094818	3.649332	0.759077
H	0.001774	1.633017	-1.635502
H	-0.401791	2.806908	0.583634
H	1.736705	-0.471626	-1.501798
C	2.677665	-2.21924	-0.661723
C	3.96007	-1.651748	-0.686346
C	2.557013	-3.577918	-0.3243
C	5.090956	-2.416839	-0.381825
H	4.070724	-0.60235	-0.942548
C	3.683068	-4.345828	-0.015017
H	1.573852	-4.043091	-0.312501
C	4.956422	-3.766519	-0.042447
H	6.075265	-1.957647	-0.409755
H	3.566853	-5.395528	0.239921
H	5.833558	-4.362014	0.193977

Table S15. Coordinates of all atoms in TS2 for CH_3CO-Asn-Phe-$NHCH_3$ in pathway 2 at B3LYP/6-31+G(d,p)

atom	coordinates		
	X	Y	Z
N	-3.797755	-1.669498	0.391481
H	-3.546383	-1.739753	1.368779
C	-2.752834	-1.858014	-0.617518
H	-2.981007	-2.761688	-1.191104
C	-2.503076	-0.654777	-1.53581
C	-1.400204	-2.050397	0.080428
H	-2.150282	-1.012716	-2.506601
H	-3.392989	-0.044547	-1.69931
C	-1.332265	0.154968	-0.914813
O	-1.077509	-3.066839	0.703325
O	-0.550664	0.783481	-1.773748
N	-1.905298	1.15399	0.111922
H	-2.277781	0.679997	0.935189
H	-2.702866	1.614928	-0.329155
N	-0.635673	-0.94027	-0.104293
H	1.991407	2.738119	0.376323
C	0.794497	-0.991284	0.234251
H	0.875951	-1.870083	0.882527
C	1.694763	-1.227196	-1.001796
C	1.2442	0.218377	1.077545
H	1.159125	-1.932064	-1.646656
O	2.083004	1.034596	0.657939
C	-5.119227	-1.736491	0.06785
O	-5.496399	-1.849583	-1.105525
C	-6.09838	-1.647635	1.219997
H	-5.615041	-1.531009	2.192438
H	-6.7652	-0.797599	1.050002
H	-6.71094	-2.55382	1.231485
N	0.70661	0.299201	2.305496
H	0.052416	-0.414886	2.594853
C	1.018729	1.374944	3.242628
H	2.097878	1.431251	3.410957
H	0.665195	2.332515	2.851731
H	0.520984	1.16014	4.188374
P	0.28248	3.914062	-0.31474
O	0.063107	3.168601	-1.70689
O	0.064134	5.399328	-0.431121
O	1.840537	3.67698	0.109169
O	-0.577163	3.162086	0.76392
H	-0.202615	2.126097	-1.672933
H	-1.153279	2.148041	0.453548
H	1.798311	-0.294517	-1.555197
C	3.054813	-1.805519	-0.656764
C	4.226018	-1.046736	-0.79839
C	3.172675	-3.132399	-0.209222
C	5.478784	-1.593946	-0.501706
H	4.15316	-0.018408	-1.139711
C	4.42124	-3.682994	0.093338
H	2.279935	-3.745035	-0.104932
C	5.58104	-2.913913	-0.051884
H	6.373022	-0.988464	-0.621387
H	4.488605	-4.712119	0.435136
H	6.552982	-3.340401	0.179148

Table S16. Coordinates of all atoms in INT3 for CH_3CO-Asn-Phe-$NHCH_3$ in pathway 2 at B3LYP/6-31+G(d,p)

atom	coordinates		
	X	Y	Z
N	-3.778784	-1.709206	0.38137
H	-3.531414	-1.875766	1.34856
C	-2.745776	-1.91459	-0.637998
H	-2.979201	-2.820046	-1.206994
C	-2.498418	-0.712756	-1.558999
C	-1.392631	-2.105353	0.060665
H	-2.149167	-1.068833	-2.531842
H	-3.386137	-0.10011	-1.721631
C	-1.31622	0.082504	-0.947975
O	-1.068555	-3.117994	0.685504
O	-0.557179	0.774866	-1.72892
N	-1.995081	1.087361	0.146671
H	-2.393246	0.611763	0.958023
H	-2.762336	1.57164	-0.321872
N	-0.631254	-0.990314	-0.1204
H	1.975093	2.737789	0.353344
C	0.797628	-1.022224	0.227882
H	0.885381	-1.89607	0.881995
C	1.707412	-1.251788	-1.001836
C	1.222739	0.20049	1.066875
H	1.179184	-1.955301	-1.6543
O	2.073486	1.01007	0.662464
C	-5.105565	-1.72292	0.060379
O	-5.48877	-1.773025	-1.114171
C	-6.07581	-1.650491	1.220784
H	-5.584033	-1.589565	2.194017
H	-6.715373	-0.773079	1.089713
H	-6.717375	-2.536144	1.198369
N	0.642564	0.300946	2.274798
H	-0.00133	-0.422074	2.56499
C	0.93121	1.388664	3.206601
H	2.000435	1.422018	3.43585
H	0.62291	2.343531	2.773307
H	0.374831	1.207101	4.126394
P	0.295882	4.004053	-0.268408
O	0.032724	3.325654	-1.725632
O	0.182071	5.503572	-0.412541
O	1.869751	3.674374	0.066272
O	-0.568855	3.273875	0.769838
H	-0.189324	2.343148	-1.679789
H	-1.324811	1.857363	0.453719
H	1.814167	-0.315843	-1.54948
C	3.065954	-1.828539	-0.647965
C	4.236895	-1.067728	-0.779443
C	3.18209	-3.156058	-0.201915
C	5.488101	-1.61373	-0.473995
H	4.165128	-0.038891	-1.119389
C	4.429031	-3.705313	0.109453
H	2.28952	-3.770228	-0.105511
C	5.588752	-2.934256	-0.025509
H	6.382355	-1.006778	-0.585675
H	4.495205	-4.734852	0.450207
H	6.559467	-3.359673	0.212523

Table S17. Coordinates of all atoms in TS3 for CH_3CO-Asn-Phe-$NHCH_3$ in pathway 2 at B3LYP/6-31+G(d,p)

atom	coordinates		
	X	Y	Z
N	4.415207	-0.146413	0.177041
H	4.24862	-0.75741	0.964889
C	3.304779	0.233172	-0.676651
H	3.733424	0.913447	-1.416878
C	2.536251	-0.914481	-1.358839
C	2.223146	1.030154	0.070218
H	2.463466	-0.73628	-2.434465
H	3.015048	-1.884375	-1.217887
C	1.081063	-0.871767	-0.82658
O	2.422301	2.078311	0.683376
O	0.109866	-1.183327	-1.579761
N	1.099834	-2.071191	0.524853
H	1.348334	-1.676261	1.431192
H	1.769805	-2.810197	0.314875
N	1.010885	0.422187	-0.100843
H	-3.077788	-1.653696	0.303111
C	-0.226069	1.144545	0.237735
H	0.115551	1.95704	0.88727
C	-0.900881	1.769541	-1.005681
C	-1.198555	0.278221	1.065795
H	-0.094596	2.142186	-1.646323
O	-2.290781	-0.097524	0.613751
C	5.690045	0.288578	-0.031048
O	5.992319	1.041334	-0.966141
C	6.727669	-0.208798	0.953784
H	6.322783	-0.887453	1.707744
H	7.519426	-0.721223	0.400245
H	7.177096	0.653154	1.455341
N	-0.791061	-0.019878	2.313263
H	0.057434	0.40469	2.662509
C	-1.591005	-0.828819	3.230649
H	-2.546594	-0.340911	3.446729
H	-1.781914	-1.811748	2.792806
H	-1.031743	-0.947232	4.158815
P	-2.326312	-3.623309	-0.317611
O	-1.685669	-3.147896	-1.745359
O	-3.042326	-4.934154	-0.544769
O	-3.481628	-2.499557	0.003901
O	-1.242638	-3.516772	0.758441
H	-1.006949	-2.425724	-1.642984
H	0.15838	-2.530154	0.60293
H	-1.425972	0.989139	-1.556557
C	-1.838547	2.914233	-0.669035
C	-3.230236	2.7708	-0.770067
C	-1.324948	4.158063	-0.264555
C	-4.086228	3.83713	-0.474852
H	-3.643587	1.815054	-1.07794
C	-2.17579	5.225373	0.035918
H	-0.248439	4.29635	-0.19256
C	-3.562217	5.068005	-0.068248
H	-5.161107	3.70501	-0.562603
H	-1.756795	6.179304	0.343894
H	-4.225655	5.896959	0.16106

Table S18. Coordinates of all atoms in PC for CH_3CO-Asn-Phe-$NHCH_3$ in pathway 2 at B3LYP/6-31+G(d,p)

atom	coordinates		
	X	Y	Z
N	-3.33194	-2.005991	-0.02672
H	-3.600263	-1.208299	0.584011
C	-2.150318	-1.871561	-0.841644
H	-2.099476	-2.756812	-1.485695
C	-2.059004	-0.582541	-1.676438
C	-0.849689	-1.860406	-0.029679
H	-2.347931	-0.707237	-2.721681
H	-2.655654	0.225741	-1.239047
C	-0.615518	-0.145069	-1.57168
O	-0.569152	-2.545525	0.937912
O	-0.07405	0.750673	-2.205581
N	-3.950866	0.35466	1.595188
H	-3.731542	0.277271	2.586133
H	-4.911127	0.687093	1.53595
N	0.015516	-0.920061	-0.599928
H	0.4942	2.985309	0.575658
C	1.391587	-0.760217	-0.084301
H	1.586496	-1.696011	0.444778
C	2.445141	-0.590397	-1.194915
C	1.399575	0.393968	0.944523
H	2.213251	-1.317571	-1.980104
O	1.6206	1.568537	0.607403
C	-4.081978	-3.136272	-0.038453
O	-3.807996	-4.125292	-0.738959
C	-5.301957	-3.124423	0.860253
H	-5.407927	-2.194614	1.422494
H	-6.194229	-3.2779	0.245908
H	-5.233547	-3.963203	1.559164
N	1.132072	0.028896	2.208041
H	0.905844	-0.940781	2.389686
C	1.037111	0.985607	3.306533
H	1.912603	1.638772	3.308675
H	0.138186	1.603321	3.216535
H	0.999284	0.430936	4.243957
P	-1.488965	3.532199	-0.256056
O	-1.069896	3.399087	-1.847959
O	-2.28721	4.808051	-0.12193
O	-0.052116	3.798471	0.499464
O	-2.053813	2.195117	0.201162
H	-0.761596	2.494469	-2.057818
H	-3.347156	1.084754	1.200112
H	2.358577	0.405971	-1.627584
C	3.852577	-0.827114	-0.679208
C	4.702664	0.248445	-0.382157
C	4.33364	-2.133196	-0.490271
C	5.9992	0.026846	0.094271
H	4.345798	1.264387	-0.525433
C	5.627492	-2.359019	-0.013107
H	3.694256	-2.980651	-0.72694
C	6.465085	-1.277718	0.282227
H	6.643521	0.872969	0.315891
H	5.982701	-3.376685	0.122107
H	7.472163	-1.45129	0.650184

Table S19. Coordinates of all atoms in RC for CH_3CO-Asn-Ile-$NHCH_3$ in pathway 1 at B3LYP/6-31+G(d,p)

atom	coordinates		
	X	Y	Z
N	-2.039779	2.192806	1.497448
H	-2.431603	2.841122	2.166157
C	-1.225639	1.092436	2.037443
H	-1.1912	1.293287	3.110415
C	-1.916002	-0.25864	1.789336
C	0.250236	1.273045	1.592329
H	-2.952685	-0.163907	2.135257
H	-1.959301	-0.47222	0.724564
C	-1.359335	-1.503977	2.464455
O	0.883619	2.198061	2.12314
C	-2.287034	2.409141	0.187458
O	-1.808763	1.678515	-0.703559
C	-3.168869	3.587385	-0.153139
H	-4.029572	3.225285	-0.722609
H	-3.522832	4.13243	0.723962
H	-2.607117	4.269105	-0.798121
H	0.199003	-0.244188	0.166065
P	-1.807947	-1.944574	-1.728631
O	-2.520827	-2.915288	-0.61429
H	-2.168694	-2.766807	0.29818
O	-2.858608	-0.676819	-1.852105
O	-1.832964	-2.686608	-3.045315
O	-0.50395	-1.41013	-1.149575
N	-0.724003	-1.379906	3.64416
H	-0.406425	-2.215346	4.117654
H	-0.625093	-0.501709	4.130242
O	-1.537456	-2.619903	1.942715
H	-2.483654	0.141138	-1.462929
N	0.781075	0.435237	0.675435
C	2.171675	0.609821	0.24201
H	2.618228	1.283684	0.977708
C	2.972356	-0.731483	0.283703
C	2.290397	1.366838	-1.096258
H	2.536026	-1.280007	1.13029
C	2.795886	-1.589879	-0.979346
C	4.45774	-0.481582	0.627149
O	3.412039	1.650345	-1.550036
H	3.294725	-1.125222	-1.837775
H	3.238215	-2.580431	-0.83526
H	1.738751	-1.726723	-1.22159
H	4.933723	0.048165	-0.205419
H	4.50664	0.192819	1.492788
C	5.24651	-1.755709	0.956629
H	5.330474	-2.423939	0.093495
H	6.263955	-1.505738	1.276834
H	4.770769	-2.318126	1.76968
N	1.15026	1.725617	-1.71593
H	0.253619	1.499773	-1.297998
C	1.158039	2.479506	-2.962379
H	1.678711	3.434742	-2.840513
H	1.656069	1.91742	-3.758648
H	0.125127	2.670934	-3.254898

Table S20. Coordinates of all atoms in TS1 for CH_3CO-Asn-Ile-$NHCH_3$ in pathway 1 at B3LYP/6-31+G(d,p)

atom	coordinates		
	X	Y	Z
N	2.292499	-2.785202	0.383646
H	2.446745	-3.754272	0.621972
C	1.076551	-2.150211	0.88062
H	0.53684	-2.948699	1.401506
C	1.385448	-1.013142	1.874832
C	0.101863	-1.6813	-0.234817
H	1.775942	-1.42662	2.811933
H	2.147154	-0.360296	1.449514
C	0.180093	-0.159377	2.206356
O	-0.040149	-2.382948	-1.265166
C	3.178865	-2.187498	-0.440125
O	3.0288	-1.008633	-0.809575
C	4.362078	-3.012091	-0.894336
H	5.282697	-2.498489	-0.602946
H	4.370119	-4.022762	-0.481224
H	4.347016	-3.072632	-1.986476
H	0.556064	0.794025	-0.725746
P	2.117403	2.366935	-0.627641
O	2.117531	2.095845	0.891698
H	1.076662	1.527163	1.617414
O	3.412864	1.626179	-1.281894
O	2.111536	3.790226	-1.114414
O	0.830493	1.578817	-1.260829
N	-0.805141	-0.654213	2.98637
H	-1.664837	-0.11021	2.960873
H	-0.954022	-1.653925	2.962727
O	0.279431	1.13732	2.211646
H	3.404346	0.656011	-1.111562
N	-0.541512	-0.581602	0.144875
C	-1.768659	-0.149664	-0.535442
H	-1.518792	0.207195	-1.547209
C	-2.865149	-1.243583	-0.75181
C	-2.312307	1.057021	0.259741
H	-2.411514	-1.948335	-1.456758
C	-3.235828	-2.033326	0.511688
C	-4.099715	-0.627908	-1.446695
O	-2.858527	0.940071	1.370299
H	-3.736643	-1.399064	1.247859
H	-3.899604	-2.867074	0.260351
H	-2.345615	-2.460884	0.982617
H	-4.644301	0.008626	-0.736304
H	-3.757697	0.033079	-2.254896
C	-5.066182	-1.660116	-2.043724
H	-5.515241	-2.296037	-1.273748
H	-5.884362	-1.162962	-2.576461
H	-4.551707	-2.313772	-2.758552
N	-2.169952	2.260797	-0.338371
H	-1.568599	2.316071	-1.149764
C	-2.561129	3.505808	0.309921
H	-3.61614	3.470118	0.593127
H	-1.968321	3.69266	1.21232
H	-2.409916	4.32589	-0.392971

Table S21. Coordinates of all atoms in INT1 for CH_3CO-Asn-Ile-$NHCH_3$ in pathway 1 at B3LYP/6-31+G(d,p)

atom	coordinates		
	X	Y	Z
N	2.168172	-2.974226	0.121051
H	2.433204	-3.946292	0.190821
C	0.886286	-2.584353	0.688013
H	0.367063	-3.51804	0.924775
C	0.942652	-1.668643	1.919491
C	-0.013156	-1.828199	-0.298291
H	0.940984	-2.220266	2.860948
H	1.842768	-1.053663	1.877966
C	-0.29181	-0.731354	1.837684
O	-0.108673	-2.079151	-1.503555
C	3.011906	-2.127213	-0.503248
O	2.732623	-0.919753	-0.645331
C	4.310814	-2.700298	-1.017315
H	5.141226	-2.161872	-0.551509
H	4.420163	-3.76773	-0.816981
H	4.367032	-2.529595	-2.096178
H	1.314277	0.566939	-1.250186
P	2.340436	2.28619	-0.365933
O	2.067858	1.866444	1.075276
H	0.786898	0.955891	1.625238
O	3.751197	1.617563	-0.868993
O	2.348949	3.74533	-0.738675
O	1.221663	1.53385	-1.338283
N	-1.318782	-1.110648	2.782556
H	-2.138951	-0.523312	2.631768
H	-1.593549	-2.079448	2.646946
O	-0.004112	0.599939	2.120412
H	3.693975	0.639345	-0.78424
N	-0.714767	-0.890614	0.384665
C	-1.722502	-0.084213	-0.339487
H	-1.218814	0.246552	-1.253501
C	-2.95452	-0.910827	-0.831248
C	-2.131238	1.183001	0.439176
H	-2.522571	-1.660944	-1.503419
C	-3.721401	-1.660847	0.266096
C	-3.871144	-0.011338	-1.690469
O	-2.944561	1.159732	1.3754
H	-4.20044	-0.971884	0.966404
H	-4.494234	-2.293211	-0.181708
H	-3.057703	-2.321729	0.831156
H	-4.407874	0.692948	-1.041866
H	-3.246162	0.596265	-2.358714
C	-4.882539	-0.784927	-2.546932
H	-5.598628	-1.342916	-1.935033
H	-5.456349	-0.098576	-3.178922
H	-4.375887	-1.500193	-3.205894
N	-1.60765	2.327622	-0.047864
H	-0.819692	2.249306	-0.683144
C	-1.853115	3.61605	0.585579
H	-2.927541	3.785772	0.687025
H	-1.399097	3.666836	1.582038
H	-1.426395	4.399361	-0.041962

Table S22. Coordinates of all atoms in INT2 for CH_3CO-Asn-Ile-$NHCH_3$ in pathway 1 at B3LYP/6-31+G(d,p)

atom	coordinates		
	X	Y	Z
N	3.858269	-1.982871	-0.62423
H	4.530246	-2.267797	-1.321467
C	2.661108	-1.300908	-1.080645
H	2.797739	-1.134217	-2.15356
C	1.329902	-2.019647	-0.810141
C	2.470098	0.087618	-0.452972
H	0.993626	-2.617838	-1.659642
H	1.429039	-2.669142	0.058547
C	0.294979	-0.919386	-0.453829
O	3.373866	0.891967	-0.218555
C	4.142162	-2.211559	0.685056
O	3.374015	-1.859706	1.590918
C	5.446856	-2.924617	0.975284
H	5.230822	-3.841766	1.530725
H	6.013905	-3.177765	0.076772
H	6.061189	-2.285174	1.615772
H	-2.284109	-1.691998	-1.655835
P	-3.869604	-1.706496	-0.153152
O	-2.791171	-2.064641	0.873903
H	-1.261663	-1.517404	0.686787
O	-4.117246	-0.081217	-0.095064
O	-5.225272	-2.364516	-0.094507
O	-3.240769	-1.968368	-1.643629
N	-0.696608	-0.769945	-1.520169
H	-1.156307	0.135965	-1.410243
H	-0.23937	-0.780318	-2.428971
O	-0.329435	-1.155333	0.772089
H	-3.293889	0.431711	0.065208
N	1.140003	0.30134	-0.269358
C	0.689033	1.584344	0.315189
H	1.341872	1.758229	1.177422
C	0.9217	2.800666	-0.645302
C	-0.762577	1.541547	0.823654
H	2.004409	2.803043	-0.810613
C	0.240817	2.672849	-2.014869
C	0.568006	4.11983	0.075217
O	-1.734881	1.406967	0.050992
H	-0.848684	2.726091	-1.934343
H	0.574162	3.479136	-2.674904
H	0.505019	1.729986	-2.502964
H	-0.521602	4.201688	0.185078
H	0.980738	4.091877	1.09244
C	1.100144	5.375565	-0.628199
H	0.648948	5.516286	-1.615517
H	0.879471	6.269945	-0.035948
H	2.187356	5.322386	-0.760199
N	-0.925496	1.780234	2.133229
H	-0.108571	1.835741	2.724594
C	-2.238142	1.848	2.766167
H	-2.888297	2.528113	2.210877
H	-2.710611	0.861312	2.801654
H	-2.111919	2.221018	3.782466

Table S23. Coordinates of all atoms in TS2 for CH_3CO-Asn-Ile-$NHCH_3$ in pathway 1 at B3LYP/6-31+G(d,p)

atom	coordinates		
	X	Y	Z
N	3.801258	-2.092833	-0.577114
H	4.383587	-2.432377	-1.328519
C	2.546981	-1.456353	-0.93113
H	2.524842	-1.411808	-2.024438
C	1.274249	-2.122872	-0.390176
C	2.439864	-0.000931	-0.436275
H	0.83243	-2.848814	-1.072916
H	1.486595	-2.615305	0.560927
C	0.321205	-0.965666	-0.077964
O	3.375817	0.790703	-0.369405
C	4.256315	-2.201982	0.699451
O	3.606908	-1.77295	1.663082
C	5.598041	-2.881396	0.877761
H	5.475154	-3.730429	1.556055
H	6.034836	-3.233613	-0.059179
H	6.288574	-2.176217	1.34958
H	-1.458885	-1.406033	-1.753274
P	-3.943054	-1.700533	-0.248014
O	-3.024865	-2.110964	1.046099
H	-2.085559	-1.810861	0.932507
O	-4.077138	-0.061575	-0.128231
O	-5.32949	-2.260453	-0.026878
O	-3.178198	-2.051136	-1.522518
N	-0.620749	-0.798946	-1.777081
H	-0.953084	0.165941	-1.760056
H	-0.084461	-0.962906	-2.626809
O	-0.575875	-1.04774	0.796112
H	-3.224474	0.424286	-0.188015
N	1.129707	0.253104	-0.126504
C	0.678705	1.559373	0.401062
H	1.182652	1.701741	1.364283
C	1.104499	2.770766	-0.501815
C	-0.842648	1.589208	0.647077
H	2.19709	2.761087	-0.473888
C	0.675224	2.644259	-1.970475
C	0.644269	4.099266	0.137874
O	-1.669874	1.454227	-0.277917
H	-0.411091	2.703799	-2.08242
H	1.123868	3.448667	-2.560605
H	1.015238	1.700682	-2.408002
H	-0.443835	4.205648	0.038112
H	0.855081	4.070003	1.214979
C	1.329511	5.338254	-0.454242
H	1.081389	5.480474	-1.510839
H	1.016969	6.241438	0.080456
H	2.420286	5.262374	-0.372676
N	-1.211124	1.923234	1.894168
H	-0.504936	1.960439	2.615154
C	-2.601883	2.12148	2.286306
H	-2.618908	2.580211	3.275067
H	-3.09985	2.783729	1.574059
H	-3.145608	1.172607	2.318906

Table S24. Coordinates of all atoms in PC for CH_3CO-Asn-Ile-$NHCH_3$ in pathway 1 at B3LYP/6-31+G(d,p)

atom	coordinates		
	X	Y	Z
N	-2.327925	-3.038968	0.3317
H	-2.312344	-3.795861	0.999939
C	-1.21506	-2.112104	0.331344
H	-0.58278	-2.368527	1.193828
C	-0.355614	-2.06667	-0.941307
C	-1.651382	-0.656773	0.558861
H	0.614171	-2.555483	-0.82305
H	-0.875588	-2.485522	-1.80729
C	-0.128586	-0.597717	-1.179154
O	-2.448756	-0.281349	1.395992
C	-3.397125	-2.904422	-0.49561
O	-3.468778	-1.966861	-1.303232
C	-4.482468	-3.952008	-0.379152
H	-4.605517	-4.44098	-1.349799
H	-4.271982	-4.710454	0.377797
H	-5.425887	-3.455353	-0.134946
H	1.750186	-2.417534	1.958286
P	3.814681	-1.006515	-0.190767
O	3.456342	-0.481172	-1.715563
H	2.490741	-0.419884	-1.860326
O	3.727125	0.361926	0.720031
O	5.27006	-1.414292	-0.204394
O	2.729384	-1.97702	0.25017
N	1.11091	-2.689211	2.712973
H	1.164239	-1.97172	3.432871
H	1.474063	-3.543985	3.12924
O	0.633854	-0.088313	-1.996766
H	2.809477	0.693297	0.840046
N	-0.91943	0.154955	-0.318174
C	-0.833457	1.626381	-0.401036
H	-0.847112	1.836419	-1.474465
C	-2.022615	2.408312	0.247982
C	0.552059	2.051871	0.133605
H	-2.893466	1.75227	0.162084
C	-1.806306	2.733704	1.735108
C	-2.33047	3.677413	-0.581222
O	1.135276	1.39816	1.015445
H	-1.023999	3.490919	1.864299
H	-2.725989	3.130733	2.173074
H	-1.527768	1.845293	2.303743
H	-1.501951	4.392809	-0.493054
H	-2.396577	3.398982	-1.640767
C	-3.634824	4.378541	-0.180239
H	-3.589953	4.784971	0.834736
H	-3.842623	5.21212	-0.859114
H	-4.484518	3.687344	-0.229666
N	1.059686	3.176639	-0.394554
H	0.580138	3.613656	-1.169089
C	2.327095	3.756701	0.043341
H	2.438581	4.732749	-0.428558
H	2.32953	3.879196	1.12914
H	3.170489	3.120363	-0.239666

Table S25. Coordinates of all atoms in RC for CH_3CO-Asn-Ile-$NHCH_3$ in pathway 2 at B3LYP/6-31+G(d,p)

atom	coordinates		
	X	Y	Z
N	-2.039779	2.192806	1.497448
H	-2.431603	2.841122	2.166157
C	-1.225639	1.092436	2.037443
H	-1.1912	1.293287	3.110415
C	-1.916002	-0.25864	1.789336
C	0.250236	1.273045	1.592329
H	-2.952685	-0.163907	2.135257
H	-1.959301	-0.47222	0.724564
C	-1.359335	-1.503977	2.464455
O	0.883619	2.198061	2.12314
C	-2.287034	2.409141	0.187458
O	-1.808763	1.678515	-0.703559
C	-3.168869	3.587385	-0.153139
H	-4.029572	3.225285	-0.722609
H	-3.522832	4.13243	0.723962
H	-2.607117	4.269105	-0.798121
H	0.199003	-0.244188	0.166065
P	-1.807947	-1.944574	-1.728631
O	-2.520827	-2.915288	-0.61429
H	-2.168694	-2.766807	0.29818
O	-2.858608	-0.676819	-1.852105
O	-1.832964	-2.686608	-3.045315
O	-0.50395	-1.41013	-1.149575
N	-0.724003	-1.379906	3.64416
H	-0.406425	-2.215346	4.117654
H	-0.625093	-0.501709	4.130242
O	-1.537456	-2.619903	1.942715
H	-2.483654	0.141138	-1.462929
N	0.781075	0.435237	0.675435
C	2.171675	0.609821	0.24201
H	2.618228	1.283684	0.977708
C	2.972356	-0.731483	0.283703
C	2.290397	1.366838	-1.096258
H	2.536026	-1.280007	1.13029
C	2.795886	-1.589879	-0.979346
C	4.45774	-0.481582	0.627149
O	3.412039	1.650345	-1.550036
H	3.294725	-1.125222	-1.837775
H	3.238215	-2.580431	-0.83526
H	1.738751	-1.726723	-1.22159
H	4.933723	0.048165	-0.205419
H	4.50664	0.192819	1.492788
C	5.24651	-1.755709	0.956629
H	5.330474	-2.423939	0.093495
H	6.263955	-1.505738	1.276834
H	4.770769	-2.318126	1.76968
N	1.15026	1.725617	-1.71593
H	0.253619	1.499773	-1.297998
C	1.158039	2.479506	-2.962379
H	1.678711	3.434742	-2.840513
H	1.656069	1.91742	-3.758648
H	0.125127	2.670934	-3.254898

Table S26. Coordinates of all atoms in TS1 for CH_3CO-Asn-Ile-$NHCH_3$ in pathway 2 at B3LYP/6-31+G(d,p)

atom	coordinates		
	X	Y	Z
N	2.292499	-2.785202	0.383646
H	2.446745	-3.754272	0.621972
C	1.076551	-2.150211	0.88062
H	0.53684	-2.948699	1.401506
C	1.385448	-1.013142	1.874832
C	0.101863	-1.6813	-0.234817
H	1.775942	-1.42662	2.811933
H	2.147154	-0.360296	1.449514
C	0.180093	-0.159377	2.206356
O	-0.040149	-2.382948	-1.265166
C	3.178865	-2.187498	-0.440125
O	3.0288	-1.008633	-0.809575
C	4.362078	-3.012091	-0.894336
H	5.282697	-2.498489	-0.602946
H	4.370119	-4.022762	-0.481224
H	4.347016	-3.072632	-1.986476
H	0.556064	0.794025	-0.725746
P	2.117403	2.366935	-0.627641
O	2.117531	2.095845	0.891698
H	1.076662	1.527163	1.617414
O	3.412864	1.626179	-1.281894
O	2.111536	3.790226	-1.114414
O	0.830493	1.578817	-1.260829
N	-0.805141	-0.654213	2.98637
H	-1.664837	-0.11021	2.960873
H	-0.954022	-1.653925	2.962727
O	0.279431	1.13732	2.211646
H	3.404346	0.656011	-1.111562
N	-0.541512	-0.581602	0.144875
C	-1.768659	-0.149664	-0.535442
H	-1.518792	0.207195	-1.547209
C	-2.865149	-1.243583	-0.75181
C	-2.312307	1.057021	0.259741
H	-2.411514	-1.948335	-1.456758
C	-3.235828	-2.033326	0.511688
C	-4.099715	-0.627908	-1.446695
O	-2.858527	0.940071	1.370299
H	-3.736643	-1.399064	1.247859
H	-3.899604	-2.867074	0.260351
H	-2.345615	-2.460884	0.982617
H	-4.644301	0.008626	-0.736304
H	-3.757697	0.033079	-2.254896
C	-5.066182	-1.660116	-2.043724
H	-5.515241	-2.296037	-1.273748
H	-5.884362	-1.162962	-2.576461
H	-4.551707	-2.313772	-2.758552
N	-2.169952	2.260797	-0.338371
H	-1.568599	2.316071	-1.149764
C	-2.561129	3.505808	0.309921
H	-3.61614	3.470118	0.593127
H	-1.968321	3.69266	1.21232
H	-2.409916	4.32589	-0.392971

Table S27. Coordinates of all atoms in INT1 for CH_3CO-Asn-Ile-$NHCH_3$ in pathway 2 at B3LYP/6-31+G(d,p)

atom	coordinates		
	X	Y	Z
N	2.168172	-2.974226	0.121051
H	2.433204	-3.946292	0.190821
C	0.886286	-2.584353	0.688013
H	0.367063	-3.51804	0.924775
C	0.942652	-1.668643	1.919491
C	-0.013156	-1.828199	-0.298291
H	0.940984	-2.220266	2.860948
H	1.842768	-1.053663	1.877966
C	-0.29181	-0.731354	1.837684
O	-0.108673	-2.079151	-1.503555
C	3.011906	-2.127213	-0.503248
O	2.732623	-0.919753	-0.645331
C	4.310814	-2.700298	-1.017315
H	5.141226	-2.161872	-0.551509
H	4.420163	-3.76773	-0.816981
H	4.367032	-2.529595	-2.096178
H	1.314277	0.566939	-1.250186
P	2.340436	2.28619	-0.365933
O	2.067858	1.866444	1.075276
H	0.786898	0.955891	1.625238
O	3.751197	1.617563	-0.868993
O	2.348949	3.74533	-0.738675
O	1.221663	1.53385	-1.338283
N	-1.318782	-1.110648	2.782556
H	-2.138951	-0.523312	2.631768
H	-1.593549	-2.079448	2.646946
O	-0.004112	0.599939	2.120412
H	3.693975	0.639345	-0.78424
N	-0.714767	-0.890614	0.384665
C	-1.722502	-0.084213	-0.339487
H	-1.218814	0.246552	-1.253501
C	-2.95452	-0.910827	-0.831248
C	-2.131238	1.183001	0.439176
H	-2.522571	-1.660944	-1.503419
C	-3.721401	-1.660847	0.266096
C	-3.871144	-0.011338	-1.690469
O	-2.944561	1.159732	1.3754
H	-4.20044	-0.971884	0.966404
H	-4.494234	-2.293211	-0.181708
H	-3.057703	-2.321729	0.831156
H	-4.407874	0.692948	-1.041866
H	-3.246162	0.596265	-2.358714
C	-4.882539	-0.784927	-2.546932
H	-5.598628	-1.342916	-1.935033
H	-5.456349	-0.098576	-3.178922
H	-4.375887	-1.500193	-3.205894
N	-1.60765	2.327622	-0.047864
H	-0.819692	2.249306	-0.683144
C	-1.853115	3.61605	0.585579
H	-2.927541	3.785772	0.687025
H	-1.399097	3.666836	1.582038
H	-1.426395	4.399361	-0.041962

Table S28. Coordinates of all atoms in INT2 for CH_3CO-Asn-Ile-$NHCH_3$ in pathway 2 at B3LYP/6-31+G(d,p)

atom	coordinates		
	X	Y	Z
N	-3.43288	-1.376138	-0.155693
H	-3.205744	-2.254462	0.302914
C	-2.271035	-0.560161	-0.489585
H	-2.220413	-0.457276	-1.580953
C	-2.358622	0.883075	0.064401
C	-1.020965	-1.353961	-0.041795
H	-1.619271	1.494311	-0.454149
H	-3.350796	1.267585	-0.195219
C	-2.141072	1.069893	1.556506
O	-1.163392	-2.49009	0.441442
O	-1.362619	1.942441	1.989314
N	-2.83766	0.276039	2.390965
H	-2.730665	0.383845	3.390609
H	-3.490566	-0.41032	2.038489
N	0.178099	-0.765612	-0.230576
H	0.241542	0.184649	-0.631019
C	1.40916	-1.495528	0.078818
H	1.078817	-2.463604	0.459682
C	2.266117	-1.778198	-1.195818
C	2.184072	-0.785578	1.19981
H	1.543986	-2.206383	-1.905522
C	2.870743	-0.518234	-1.832786
C	3.328446	-2.862033	-0.910959
O	2.376195	0.443695	1.221716
H	3.67981	-0.111997	-1.21542
H	3.288384	-0.754275	-2.815985
H	2.123075	0.267931	-1.967582
H	4.093471	-2.45918	-0.234017
H	2.851297	-3.696643	-0.379827
C	4.009914	-3.415052	-2.169719
H	4.596996	-2.650997	-2.688719
H	4.690945	-4.232088	-1.909109
H	3.270817	-3.809348	-2.877336
C	-4.62396	-1.242695	-0.80193
O	-4.84073	-0.316797	-1.5959
C	-5.680696	-2.277411	-0.475569
H	-5.352573	-3.011391	0.263481
H	-6.571933	-1.766464	-0.100288
H	-5.959641	-2.796907	-1.397104
N	2.666843	-1.582536	2.16966
H	2.460065	-2.570643	2.139841
C	3.469182	-1.076053	3.27853
H	2.890969	-0.375313	3.887888
H	3.774773	-1.920591	3.895915
H	4.358908	-0.561045	2.906035
P	0.89566	3.177771	-0.83522
O	-0.075686	3.738602	0.362554
O	1.158463	4.340527	-1.76441
O	0.309987	1.875174	-1.382195
O	2.305216	2.828718	-0.07465
H	-0.494785	3.030202	0.912419
H	2.289629	1.925038	0.320381

Table S29. Coordinates of all atoms in TS2 for CH_3CO-Asn-Ile-$NHCH_3$ in pathway 2 at B3LYP/6-31+G(d,p)

atom	coordinates		
	X	Y	Z
N	-3.579746	-1.1065	-0.174495
H	-3.404352	-2.092898	-0.331735
C	-2.430398	-0.231625	-0.36465
H	-2.58433	0.362697	-1.273972
C	-2.127277	0.712753	0.81515
C	-1.186595	-1.121416	-0.564443
H	-1.894342	1.712185	0.447475
H	-2.98082	0.7959	1.492032
C	-0.915125	0.207813	1.595147
O	-1.291927	-2.236272	-1.105737
O	-0.048723	1.077431	2.088855
N	-1.149458	-0.827099	2.473625
H	-0.353652	-1.136998	3.017218
H	-1.760764	-1.574364	2.172358
N	-0.098907	-0.543164	-0.012633
H	0.064149	1.138112	-1.0474
C	1.105886	-1.379053	0.133138
H	0.817661	-2.286993	0.682783
C	1.737583	-1.900017	-1.208386
C	2.160297	-0.637755	0.97214
H	0.942589	-2.496955	-1.663592
C	2.132309	-0.802146	-2.205271
C	2.911974	-2.856775	-0.901899
O	2.655847	0.455692	0.644075
H	2.968424	-0.200429	-1.836674
H	2.433346	-1.253403	-3.155726
H	1.299742	-0.12752	-2.417538
H	3.768652	-2.285762	-0.518617
H	2.613561	-3.543532	-0.098383
C	3.364824	-3.694587	-2.105609
H	3.774369	-3.073984	-2.908841
H	4.1452	-4.403494	-1.808695
H	2.5297	-4.271785	-2.520205
C	-4.857986	-0.648857	-0.200717
O	-5.121058	0.561695	-0.254437
C	-5.948082	-1.699211	-0.134659
H	-5.562573	-2.720178	-0.090451
H	-6.564114	-1.513258	0.749956
H	-6.59055	-1.5963	-1.013943
N	2.576622	-1.275484	2.082915
H	2.199279	-2.187838	2.295193
C	3.631191	-0.748574	2.942247
H	3.365709	0.248161	3.304049
H	3.749927	-1.419621	3.792901
H	4.580325	-0.680712	2.401544
P	0.793805	3.196562	-0.71812
O	0.272289	3.15613	0.72959
O	0.648382	4.471608	-1.503119
O	0.051018	1.990253	-1.545828
O	2.365678	2.761527	-0.722361
H	0.079048	1.920244	1.490677
H	2.493522	1.898231	-0.255927

Table S30. Coordinates of all atoms in PC for CH_3CO-Asn-Ile-$NHCH_3$ in pathway 2 at B3LYP/6-31+G(d,p)

atom	coordinates		
	X	Y	Z
N	-2.582738	-2.194836	-0.516952
H	-2.133324	-2.704947	-1.267077
C	-1.782106	-1.19374	0.165415
H	-2.241056	-0.205774	0.036217
C	-1.493543	-1.435156	1.649078
C	-0.389084	-1.124265	-0.465045
H	-2.233704	-0.998608	2.319936
H	-1.435752	-2.510407	1.842846
C	-0.10673	-0.800527	1.897987
O	-0.174234	-1.264771	-1.672531
O	-0.128032	0.531188	2.32107
N	0.606439	-1.517901	2.920314
H	1.429766	-1.013298	3.234983
H	0.864618	-2.455169	2.628072
N	0.51895	-0.895655	0.5172
H	-0.526292	1.53687	-1.505709
C	1.931451	-0.575096	0.328143
H	2.377041	-0.72785	1.314782
C	2.693591	-1.545187	-0.656141
C	2.124606	0.916111	-0.004328
H	2.026492	-2.403223	-0.789085
C	2.969958	-0.943019	-2.042941
C	3.982943	-2.065384	0.020084
O	1.291977	1.575652	-0.65497
H	3.717844	-0.142905	-1.990499
H	3.357886	-1.711451	-2.717365
H	2.05493	-0.544097	-2.483179
H	4.68078	-1.230335	0.176588
H	3.725601	-2.446479	1.016856
C	4.700326	-3.176611	-0.756407
H	5.096588	-2.82263	-1.71299
H	5.542819	-3.565771	-0.175062
H	4.021915	-4.013363	-0.961999
C	-3.928704	-2.278424	-0.374142
O	-4.549588	-1.565758	0.428848
C	-4.634579	-3.303298	-1.239147
H	-3.958574	-3.859847	-1.892135
H	-5.163622	-4.007996	-0.591152
H	-5.3819	-2.79103	-1.852091
N	3.273911	1.457805	0.429851
H	3.884239	0.903966	1.014327
C	3.662993	2.836799	0.149262
H	3.085268	3.543168	0.754197
H	4.720957	2.95156	0.384898
H	3.501959	3.063209	-0.906905
P	-1.63425	2.976865	-0.292699
O	-1.761609	2.056847	0.921036
O	-2.760657	3.922876	-0.617038
O	-1.345817	2.055318	-1.628507
O	-0.24859	3.856179	-0.161514
H	-0.713596	1.092182	1.733051
H	0.522603	3.251911	-0.195962

Table S31. Electronic and zero-point energies for CH_3CO-Asn-Phe-$NHCH_3$ in pathway 1 at MP2/6-311+G(2d,2p)//B3LYP/6-31+G(d,p).

	Zero-point correction at B3LYP/6-31+G(d,p)	Single-point Energy at MP2/6-311+G(2d,2p)	Sum of single-point and zero-point energies
RC	0.417409	-1783.1684623	-1782.751053
TS1	0.415921	-1783.1411383	-1782.725217
INT1	0.419836	-1783.1708978	-1782.751062
INT2	0.419658	-1783.1692603	-1782.749602
TS2	0.414783	-1783.1539508	-1782.739168
INT3	0.419554	-1783.1590346	-1782.739481
TS3	0.419554	-1783.1590346	-1782.739481
PC	0.417603	-1783.1508407	-1782.733238

Table S32. Electronic and zero-point energies for CH_3CO-Asn-Phe-$NHCH_3$ in pathway 2 at MP2/6-311+G(2d,2p)//B3LYP/6-31+G(d,p).

	Zero-point correction at B3LYP/6-31+G(d,p)	Single-point Energy at MP2/6-311+G(2d,2p)	Sum of single-point and zero-point energies
RC	0.418005	-1783.1877234	-1782.769718
TS1	0.416341	-1783.1470882	-1782.730747
INT1	0.41784	-1783.1549852	-1782.737145
INT2	0.418925	-1783.1629462	-1782.744021
TS2	0.417596	-1783.1473702	-1782.729774
INT3	0.418283	-1783.1519411	-1782.733658
TS3	0.412225	-1783.1453193	-1782.733094
PC	0.415822	-1783.1774084	-1782.761586

Table S33. Electronic and zero-point energies for CH_3CO-Asn-Phe-$NHCH_3$ in pathway 1 at M06-2X/6-31+G(d,p)// B3LYP/6-31+G(d,p).

	Zero-point correction at B3LYP/6-31+G(d,p)	Single-point Energy at M06-2X/6-31+G(d,p)	Sum of single-point and zero-point energies	Relative energy / kJ mol^{-1}
RC	0.417409	-1786.1681225	-1785.750714	0
TS1	0.415921	-1786.1407509	-1785.724830	68.0
INT1	0.419836	-1786.1697156	-1785.749880	2.19
INT2	0.419658	-1786.1682016	-1785.748544	5.70
TS2	0.414783	-1786.1541696	-1785.739387	29.6
INT3	0.419554	-1786.1559928	-1785.736439	37.5
TS3	0.419554	-1786.1559929	-1785.736439	55.4
PC	0.417603	-1786.1472088	-1785.729606	17.7

Table S34. Electronic and zero-point energies for CH_3CO-Asn-Phe-$NHCH_3$ at M06-2X/6-31+G(d,p)// B3LYP/6-31+G(d,p).

	Zero-point correction at B3LYP/6-31+G(d,p)	Single-point Energy at M06-2X/6-31+G(d,p)	Sum of single-point and zero-point energies	Relative energy / kJ mol^{-1}
RC	0.418005	-1786.1874733	-1785.769468	0
TS1	0.416341	-1786.1463023	-1785.729961	103
INT1	0.41784	-1786.1529396	-1785.735100	90.3
INT2	0.418925	-1786.1627460	-1785.743821	67.4
TS2	0.417596	-1786.1507944	-1785.733198	95.3
INT3	0.418283	-1786.1496534	-1785.731370	100
TS3	0.412225	-1786.1430695	-1785.730845	101
PC	0.415822	-1786.1711373	-1785.755315	37.2

Table S35. Electronic and zero-point energies for CH_3CO-Asn-Ile-$NHCH_3$ in pathway 1 at MP2/6-311+G(2d,2p)//B3LYP/6-31+G(d,p).

	Zero-point correction at B3LYP/6-31+G(d,p)	Single-point Energy at MP2/6-311+G(2d,2p)	Sum of single-point and zero-point energies
RC	0.421404	-1670.3302246	-1669.908821
TS1	0.418699	-1670.2911433	-1669.872444
INT1	0.422974	-1670.3211724	-1669.898198
INT2	0.423269	-1670.3208526	-1669.897584
TS2	0.421249	-1670.3029444	-1669.881695
PC	0.418413	-1670.3214783	-1669.903065

Table S36. Electronic and zero-point energies for CH_3CO-Asn-Ile-$NHCH_3$ in pathway 2 at MP2/6-311+G(2d,2p)//B3LYP/6-31+G(d,p).

	Zero-point correction at B3LYP/6-31+G(d,p)	Single-point Energy at MP2/6-311+G(2d,2p)	Sum of single-point and zero-point energies
RC	0.421681	-1670.3369357	-1669.915255
TS1	0.41946	-1670.2952413	-1669.875781
INT1	0.421704	-1670.3169406	-1669.895237
INT2	0.421344	-1670.3178159	-1669.896472
TS2	0.419559	-1670.2915147	-1669.871956
PC	0.417962	-1670.3205059	-1669.902544

Table S37. Electronic and zero-point energies for CH_3CO-Asn-Ile-$NHCH_3$ in pathway 1 at M06-2X/6-31+G(d,p)// B3LYP/6-31+G(d,p).

	Zero-point correction at B3LYP/6-31+G(d,p)	Single-point Energy at M06-2X/6-31+G(d,p)	Sum of single-point and zero-point energies	Relative energy / kJ mol^{-1}
RC	0.421404	-1673.0950250	-1672.673344	0
TS1	0.418699	-1673.0577063	-1672.638246	92.2
INT1	0.422974	-1673.0855152	-1672.663811	25.0
INT2	0.423269	-1673.0862929	-1672.663024	27.1
TS2	0.421249	-1673.0659465	-1672.644698	75.2
PC	0.418413	-1673.0794457	-1672.661033	32.3

Table S38. Electronic and zero-point energies for CH_3CO-Asn-Ile-$NHCH_3$ in pathway 2 at M06-2X/6-31+G(d,p)// B3LYP/6-31+G(d,p).

	Zero-point correction at B3LYP/6-31+G(d,p)	Single-point Energy at M06-2X/6-31+G(d,p)	Sum of single-point and zero-point energies	Relative energy / kJ mol^{-1}
RC	0.418005	-1786.1874733	-1785.769468	0
TS1	0.416341	-1786.1463023	-1785.729961	106
INT1	0.41784	-1786.1529396	-1785.735100	53.5
INT2	0.418925	-1786.1627460	-1785.743821	49.5
TS2	0.417596	-1786.1507944	-1785.733198	121
PC	0.415822	-1786.1711373	-1785.755315	50.7
